# The time course of subsequent hospitalizations and associated costs in survivors of an ischemic stroke in Canada

**DOI:** 10.1186/1472-6963-6-99

**Published:** 2006-08-14

**Authors:** J Jaime Caro, Kristen Migliaccio-Walle, Khajak J Ishak, Irina Proskorovsky, Judith A O'Brien

**Affiliations:** 1 Caro Research Institute, Concord, MA, USA; 2 Division of General Internal Medicine, McGill University, Montreal, Quebec, Canada; 3 Caro Research Institute, Montreal, Quebec, Canada

## Abstract

**Background:**

Documentation of the hospitalizations rates following a stroke provides the inputs required for planning health services and to evaluate the economic efficiency of any new therapies.

**Methods:**

Hospitalization rates by cause were examined using administrative data on 18,695 patients diagnosed with ischemic stroke (first or subsequent, excluding transient ischemic attack) in Saskatchewan, Canada between 1990 and 1995. Medical history was available retrospectively to January 1980 and follow-up was complete to March 2000. Analyses evaluated the rate and timing of all-cause and cardiovascular hospitalizations within discrete periods in the five years following the index stroke. Cardiovascular hospitalizations included patients with a primary diagnosis of ischemic stroke, transient ischemic attack, myocardial infarction, stable or unstable angina, heart failure or peripheral arterial disease.

**Results:**

One-third (36%) of patients were identified by a hospitalized stroke. Mean age was 70.5 years, 48.0% were male, half had a history of stroke or a transient ischemic attack at the time of their index stroke. Three-quarters of the patients (72.7%) were hospitalized at least once during a mean follow-up of 4.6 years, accruing CAD $24 million in the first year alone. Of all hospitalizations, 20.4% were related to cardiovascular disease and 1.6% to bleeds. In the month following index stroke, 12.5% were admitted, an average of 1.04 times per patient hospitalized. Strokes accounted for 33% of all hospitalizations in the first month. The rate diminished steadily throughout the year and stabilized in the second year when approximately one-third of patients required hospitalization, at a rate of about one hospitalization for every two patient-years. Mean lengths of stay ranged from nine days to nearly 40 days. Close-fitting Weibull functions allow highly specific probability estimates. Other cardiovascular risk factors significantly increased hospitalization rates.

**Conclusion:**

After stroke, there are frequent hospitalizations accounting for substantial additional costs. Though these rates drop after one year, they remain high over time. The number of other cardiovascular causes of hospitalization confirms that stroke is a manifestation of disseminated atherothrombotic disease.

## Background

There has been increasing recognition that health policy founded on methodologically sound analyses is of vital concern for clinicians [1]. As better methods of prevention, diagnosis, treatment and rehabilitation become available, it is important to carry out these evaluations that go beyond the clinical trials to provide estimates of the relative returns of the new interventions and help policy makers discriminate what is useful and affordable [2]. Outdated or incomplete information is a major problem for those trying to inform the authorities who make decisions regarding the use of limited health care resources. Assembling these inputs is not an easy task and failure to do so can lead to serious inaccuracies in the modeled outcomes, and thus, in the messages communicated [3]. Therefore, it is important to examine and report on the use of resources, particularly those playing a major role in the management of these patients. These reports must be detailed enough to enable analysts to adjust the measures to their own context and, in the context of any chronic illness, time-dependency is one of the crucial details.

For stroke, these evaluations involve modeling the patients' course over time and accounting for all of the consequences and their costs. While the initial management of stroke is reasonably well documented [4,5], the ensuing resource use is much less so [6]. A key component of this is the expense for admissions to hospital following a stroke. Ideally, the long-term hospitalization rates are obtained from long-term follow-up studies, but although several have been reported [7-9], none of them provide the information in a form that can be used by other researchers to evaluate new interventions in other locales. Rather than estimates of the costs per se (which vary from place to place and with time), what is required are the resource use rates. Hospitalization rates have been published for the Medicare population in the United States (US) [10], but these are cross-sectional, not given as functions of time since stroke and other determinants.

Thus, the objective of this work was to provide updated, comprehensive information on the rates of hospitalization following an ischemic stroke. In this paper, we present a detailed analysis of hospitalizations over the first five years. While this does not address the economic efficiency of any one intervention, it is the information required for such analyses. We provide the information in a manner that enables other analysts to incorporate it in economic models of the implications of new treatments.

## Methods

Data were obtained from Saskatchewan Health, a provincial government department that oversees ten health care databases [11], including outpatient prescriptions and physician services, hospitalizations, and vital statistics for approximately one million residents covered by health insurance (excluding indigenous people [12]). All adults (21 years or older) diagnosed with an ischemic stroke between January 1, 1990 and December31, 1995 were sought. To achieve a comprehensive enumeration of these patients, it was decided to take records from both the hospital services file and the physician services file because the first notice of a stroke may appear in either file as the physician in the emergency room may be the first one to record the diagnosis; and also some patients with stroke may not be hospitalized. This analysis was part of a larger study of patients with atherothrombosis [13]. The first of ischemic stroke (ICD-9 codes 433, 434, 436, and 362.3), myocardial infarction, or peripheral arterial disease documented during the study period was the index diagnosis and its first recording in either file was the index date. History was available to January 1, 1980 and follow-up through March 2000.

Medical history prior to the index diagnosis was examined using data from the hospital services file and the physician services file complemented by the prescription file to identify risk factors. Risk factors of interest were based primarily on ICD-9 codes recorded in the hospital and physician services files: atrial fibrillation, angina, diabetes mellitus, heart failure, treatment of hypercholesterolemia (based on documentation of a prescription), hypertension, prior ischemic stroke, myocardial infarction, and transient ischemic attack. Data on smoking history or laboratory values were not available in these data bases.

The risk and timing of first subsequent hospitalization following the index stroke was estimated using standard failure time techniques [14]. A subsequent hospitalization was considered if it began at least seven days beyond the index date to avoid counting the initial hospitalization following a physician-recorded diagnosis, and to avoid inter-hospital transfers for the first acute hospitalization. The hospitalizations were classified as due to cardiovascular disease if the primary diagnosis was ischemic stroke, transient ischemic attack, myocardial infarction, stable or unstable angina, heart failure or peripheral arterial disease. Given that many of these patients receive anticoagulants or aspirin, hospitalizations due to bleeds (intracranial hemorrhage, gastrointestinal hemorrhage or unspecified hemorrhage) were also examined separately. As many patients are hospitalized more than once, all hospitalizations were considered using three additional measures (i.e., frequency, hazard, total rate) in each period – days 1–30, 31–60, 61–180, 181–365 – and annually thereafter. Frequency is expressed as hospitalizations per patient hospitalized (per month), hazard is expressed as patients hospitalized per patient year, and the total rate is expressed as hospitalizations per patient year.

Parametric Weibull regression analyses [14,15], were used to fit these rates as functions of time. A two-parameter Weibull was chosen because it is a very flexible distribution that fit the decreasing rates over time very well. The shape parameter indicates the slope of the curve while the scale parameter provides a starting point. Using multivariate Cox proportional hazards [16,17] the effect of potential risk factors (age over 65 years, sex, hypertension, diabetes, hypercholesterolemia, atrial fibrillation, heart failure, angina, prior transient ischemic attack, ischemic stroke, or myocardial infarction) was examined, using the same units of analysis as for the crude estimates. Rates of hospitalization were estimated from the index date to the date of death, patient censoring or the last follow-up date available.

The costs of a hospitalization were obtained from the Ontario Case Cost Project Case Mix Group [18,19] in 2002 Canadian dollars (CAD $1 = USD $0.65 = €0.70 on June 1, 2002). As costs do not translate well among countries, a more applicable "relative value weight" was created by dividing the hospitalization cost by the per diem for myocardial infarction (CAD $566.59, USD$368.28, €396.61). No discounting was applied, as there is no comparative analysis with differential timing.

## Results

Of 18,704 patients (Table 1) with index stroke (nine excluded for administrative reasons), 60.2% died and 3.1% emigrated during follow-up. Patients were predominantly elderly and most had one or more risk factors. There were 53,406 hospitalizations over 86,153 person-years of followup (mean 4.6 years), a hospitalization hazard of 62.0/100 person-years. Most patients (72.7%) were hospitalized at least once, with mean time to first hospitalization 1.59 years (sd 1.97, median 0.74); mean length of stay 13.9 days (median 5, range 1 to >180, standard deviation 51.7).

At the time of index stroke, 21.3% of patients had previously filled at least one prescription for an ACE inhibitor, 30.6% for a beta blocker, 23.4% a calcium channel blocker, 4.4% a lipid lowering agent (e.g., statin), 37.3% aspirin, and less than 1% each for dipyridamole or ticlopidine. Following the index stroke, 32.9% of patients filled at least one prescription for an ACE inhibitor, 16.9% beta blocker, 26.1% calcium channel blocker, 8.4% lipid lowering agent, 36.0% aspirin, 4.5% ticlopidine, 1.1% clopidogrel, and less than 1% dipyridamole.

Stroke and transient ischemic attack accounted for 10.7% of subsequent hospitalizations, other cardiovascular disease for 9.7%, and hemorrhage for 1.6%. The majority of hospitalizations (78%) were for unrelated diagnoses, though in the first six months, cardiovascular disease was common (43.0%). Most admissions were from emergency (38.9%) or classified "urgent" (31.5%), especially for hemorrhage (80.4%). Stays ranged from a low of 8.8 (± 25.3) days if discharged home to a high of 43.5 (± 138.0) among patients who died; Hospitalizations for ischemic stroke (39.6 ± 117.0 days) or intracranial hemorrhage (35.8 ± 88.9 days) were longest while other cardiovascular disease (11.8 ± 32.6 days) and other bleeds (10.5+29.5 days) were much lower; transient ischemic attacks were the shortest at 9.0 (± 36.3) days.

In the month following index stroke, 12.5% were admitted, an average of 1.04 times per patient hospitalized (Table 2). The rate diminished steadily throughout the year and stabilized in the second year when approximately one-third of patients required hospitalization, at a rate of about one hospitalization for every two patient-years. The resulting equation for the total rate (TR) of hospitalizations over time follows a Weibull distribution (r^2 ^= 0.95):

*TR *= 2.82*t*^-0.2394^

This equation can be used to derive the probability of hospitalization at any point in time. For example, beginning at day 366 following stroke, the expected total rate is 0.69 per year; so in the following month the probability of being hospitalized is about 0.057. After three years, this drops to 0.044 (inthefirst month of the fourth year).

The rate for cardiovascular disease (Table 3) followed the same Weibull pattern with *A *= 1.88, *B *= -0.4263 (r^2 ^= 0.96). Subsequent strokes accounted for 33.0% of hospitalizations in the first month, transient ischemic attacks for 5.8% (Table 4), with Weibull functions: *A *= 2.86, *B *= -0.6552 (r^2 ^= 0.98) for stroke; *A *= 0.274, *B *= -0.4539 (r^2 ^= 0.96) for transient ischemic attack. Rates of hospitalizations related to non-cerebrovascular disease (i.e., myocardial infarction, angina) were considerably lower at all points in time (range: 0.01–0.02 per patient year).

### Predictors

The risk of hospitalization was increased by 36.2% (95% CI: 30.3%–42.4%) if older than 65 years; by 18.1% (14.1%–22.2%) in men; by 20.5% (15.8%–25.3%) in diabetics; by 20.9% (13.9%–28.4%) with atrial fibrillation; by 21.6% (16.6%–26.8%) with heart failure; by 12.1% (7.6%–16.8%) with angina; by 10.1% (2.7%–14.8%) with prior myocardial infarction; by 14.0% (9.7%–18.4%) among hypertensives; by 4.0% (0.2%–8.1%) with previous transient ischemic attack. History of stroke decreased the risk of hospitalization by 8.0% (4.2%–11.7%). Hypercholesterolemia was not a significant predictor.

These effects were more pronounced if only cardiovascular disease hospitalizations were considered: 67.2% (55.0%–80.5%) increase if older than 65 years; 34.3% (27.3%–41.7%) in men; 40.8% (32.8%–49.2%) in diabetics; 38.4% (27.4%–50.3%) with atrial fibrillation; 35.9% (27.9%–44.5%) with heart failure; 23.1% (15.9%–30.8%) with angina; 24.1% (14.8%–34.0%) with prior myocardial infarction; and 36.1% (27.7%–45.0%) among hypertensives. Neither prior stroke nor transient ischemic attack was significantly associated with risk of cardiovascular disease hospitalization, however. The impact of hypercholesterolemia remained unchanged.

### Costs

In the year following index stroke, the 18,695 patients accumulated CAD $24 million (USD $15.6, €16.8 in 2002) in hospitalizations due to cardiovascular disease. This is equivalent to a relative value weight of 2.3 per diems per patient, though many patients add little cost because they die (23% by one year). These hospitalizations are only a fraction of the total resource consumption. In subsequent years, the cost due to these hospitalizations dropped to a relative value of 0.95 perdiem/patient/year; also influenced by the ongoing high mortality (50% by year five). The occurrence of bleeds requiring hospitalization is much lower but still adds CAD $5 million (USD $3.25, €3.5 in 2002) in year one; remaining fairly steady at a relative value of 0.17 per diems/patient/year.

**Figure 1:**
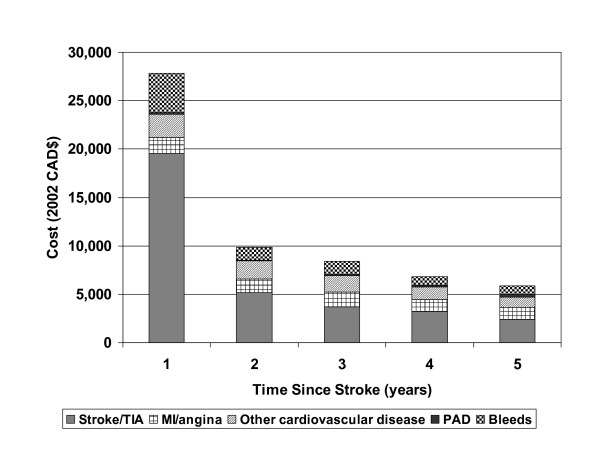
Costs due specific hospitalizations, accumulated each year after stroke in 18,695 patients, by reason for admission.

The pattern of costs varies somewhat from year to year (Figure 1). They are not only highest in the first year after diagnosis, but they are also largely due to stroke or transient ischemic attack. In subsequent years the amounts due to other cardiovascular disease and bleeds remain in absolute terms about the same, but grow in importance because stroke/transient ischemic attack drops considerably.

## Discussion

In this study, we analyzed a large administrative database to estimate the hospitalization rates following an ischemic stroke, their time course, and the ensuing costs. This information can be used to carry out budget impact and other economic analyses required by policy makers to address the appropriateness and affordability of novel interventions [2,3]. While costs of stroke have been documented often [20] and some studies of the post-stroke course have recently been published [21,22], to our knowledge ours is the first to provide a detailed description of hospitalizations following an ischemic stroke in an unrestricted (i.e., no exclusion criteria) population with long-term follow-up. This study was comparable to other published studies in terms of the proportion of patients suffering a secondary event (72.7% vs 76.7%) [21] and medication use post index event [22].

We have provided functions to estimate the rate at any time after a stroke, by cause of hospitalization, so they can be used in economic models [23,24]. Transition probabilities in Markov models or recurrence frequencies in more sophisticated discrete event simulations can be easily calculated from the hazards. For settings where it is felt that the rates based on Saskatchewan data may not apply directly, the estimates can be recalibrated by using the periodto-period ratios and applying these to a known rate for a specific period in the setting at issue.

Hospitalization, particularly for cardiovascular problems, is most frequent in the first six months following the stroke, highlighting the importance of action plans to prevent these complications in stroke survivors. From year two onward, less than half of the hospitalizations are due to cardiovascular disease and even fewer because of subsequent strokes or transient ischemic attacks. The likelihood of hospitalization (both all-cause or for cardiovascular disease) is highest among patients over 65 years of age. Atrial fibrillation at the time of the stroke is also a key factor in determining these hospitalizations. Presence of heart failure greatly increases the risk of all-cause hospitalization, whereas, diabetes plays a major role in predicting hospitalization due to cardiovascular disease. Other important risk factors included male gender, prior myocardial infarction, and hypertension. Prior stroke was protective of all-cause hospitalization in this analysis. Although perhaps counterintuitive, this is likely a marker of a select, healthier population who survived an earlier event long enough to suffer a subsequent stroke and qualify for this analysis.

The rates estimated here consider the hospitalizations without attribution to the stroke per se. Thus analysts must be cautious about imputing all hospitalizations to the preceding stroke – many would likely occur regardless given the age of these patients and other conditions they have. Whether stroke aggravates the severity of those other conditions or in some way leads to increased rates of unrelated hospitalizations cannot be determined from this study. Nevertheless, comparison with other conditions suggests that stroke does lead to an increase in hospitalizations. For example, in the same Saskatchewan population [25], the rate of hospitalization for cardiovascular disease in the month following a diagnosis of peripheral arterial disease was half that documented here. After a myocardial infarction, the initial rates were higher. Nevertheless, in both these other conditions, the rates in years two and beyond are very similar to that after stroke, suggesting that the effects are relatively acute and then the underlying age and general condition of patients takes over.

Use of administrative databases such as those of Saskatchewan Health has some limitations. Definition of the populations and identification of events is dependant on accuracy of the ICD-9 codes submitted to Saskatchewan Health. For identifying cases of stroke, two of the codes used (434, 436) have been previously validated [26-29]. With respect to identifying outcome events, validation work has indicated a low error rate overall [11] and this is even lower for cardiovascular events. The observed lengths of stay associated with hospitalizations for transient ischemic attack, however, may indicate potential errors in coding given their duration. A study from the Canadian province of Alberta estimated that 30% of transient ischemic attacks reported in the Calgary region were incorrectly coded [30], indicating that the majority of admissions are coded correctly. Given the comprehensive health insurance, and lack of other facilities, all residents seek care within the system. Moreover, given the global hospital budgets extant in Canada, there is no reimbursement incentive to alter coding. Although the majority of index strokes in this study were first recorded in the physician file, this does not imply that most of these patients were not hospitalized. Rather it results from the way cases are recorded in the databases, with the physician claims in one set and the hospital records in another. As patients with stroke may first be seen by a physician in the emergency room or outpatient clinic, this "visit" becomes the index recording rather than the acute hospitalization. This should not affect in any way the interpretation of the results of this study.

Another limitation to administrative data is that they do not contain clinical information such as results of laboratory tests, smoking, family history, and blood pressure and so on. This confines the set of predictive factors to the information that appears on billing records or is stored in other linked data sets. The factors identified may, thus, be carrying part of the effect of clinical determinants and might not be as strong predictors if these latter ones were to be included.

## Conclusion

Despite these limitations, the data we obtained from Saskatchewan Health provide a wide-ranging, novel assessment of a large cohort of patients diagnosed with a stroke over five years. These estimates can provide inputs to analyses of the economic efficiency of new stroke therapies and a basis for future studies of this type. It is clear from our findings that patients who have had a stroke are expensive to manage, with frequent, costly hospitalizations, which place a significant burden on them, their families and the health care system.

## Abbreviations

CI: Confidence Interval

ICD-9: International Classification of Diseases, version 9

MI: Myocardial Infarction

PAD: Peripheral Arterial Disease

PY: Person Years

TIA: Transient Ischemic Attack

## Competing interests

This work was supported in part by a grant from Sanofi-Synthelabo and Bristol-Myers Squibb to Caro Research. The grantors collaborated in helping set the specifications for the analyses but had no role in methodological decisions or interpretation of results. They were also allowed to review and comment on this manuscript but were explicitly forbidden from exerting any editorial control. Expenses for travel to present the findings at the ISPOR Annual Meeting, Arlington, VA, 2003 were reimbursed.

## Authors' contributions

Jaime Caro designed the study, participated in the data analyses and writing of the paper. Kristen Migliaccio-Walle helped design the study and write the paper. Khajak J. Ishak led the analyses and participated in writing the paper. Irina Proskorovsky conducted the analyses and helped write the paper. Judith A. O'Brien provided the cost estimates and participated in writing the paper. All authors read and approved the final manuscript.

## Pre-publication history

The pre-publication history for this paper can be accessed here:



## Figures and Tables

**Table 1:** Patient characteristics, medical history, and principal diagnosis

**Item**	**Result**
Age (mean ± standard deviation, years)	70.5 ± 9.9
Male	8,980 (48)*
Qualifying diagnosis in hospital	6,807 (36)
Medical History	
Atrial Fibrillation	1,812 (10)
Angina	4,848 (26)
Heart Failure	5,319 (28)
Diabetes Mellitus	4,485 (24)
Hypercholesterolemia	1,183 (6)
Hypertension	12,597 (67)
Ischemic Stroke	4,405 (24)
Myocardial Infarction	2,209 (12)
Transient Ischemic Attack	5,150 (28)
Identifying ICD-9 code	
436 (acute, but ill-defined cerebrovascular disease)	83.2%
434.9 (occlusion of cerebral arteries, unspecified)	8.4%
434 (occlusion of cerebral arteries)	3.2%
433.1 (occlusion and stenosis of precerebral arteries, carotid artery)	1.8%
434.1 (occlusion of cerebral arteries, cerebral embolism)	1.5%
Other (362.3. 433.2–9)^†^	1.9%

* number (%)

^†^Retinal vascular occlusion, Occlusion and stenosis of precerebral arteries, vertebral artery, multiple and bilateral, other specified precerebral artery, unspecified precerebral artery (NOS)

**Table 2:** All cause hospitalizations among patients diagnosed with ischemic stroke according to time since index stroke

	**At Risk**		**All Cause Hospitalization**					
		
**Time Period**	N*	PY**	**Patients**	**Hosp**^†^	**%**^‡^	**Freq**^§^	**Hazard**^#^	**Total Rate°**
Month 1	18,695	1,429	2,338	2,440	12.5	1.04	1.64	1.71
Month 2	16,821	1,359	1,375	1,455	8.2	1.06	1.01	1.07
Months 3–6	16,319	5,274	3,325	4,386	20.4	0.44	0.63	0.83
Months 7–12	15,361	7,457	3,436	4,870	22.4	0.23	0.46	0.65
Year 2	14,436	13,642	4,654	7,935	32.2	0.14	0.34	0.58
Year 3	12,917	12,265	3,992	6,903	30.9	0.14	0.33	0.56
Year 4	11,628	11,081	3,611	5,948	31.1	0.14	0.33	0.54
Year 5	10,514	9,997	3,269	5,351	31.1	0.14	0.33	0.54

*N=number surviving to beginning of each time period

**PY=person years accumulated by patients during each period

^†^Hospitalization count in each period

^‡^% gives the proportion of patients hospitalized

^§^Frequency expressed as hospitalizations per patient hospitalized (per month)

^#^Hazard expressed as patients hospitalized per patient year

°Total rate expressed as hospitalizations per patient year

**Table 3:** Cardiovascular disease-related hospitalizations among patients diagnosed with ischemic stroke according to time since index stroke

	**At Risk**		**Hospitalization Due to Cardiovascular Disease**					
		
**Time Period**	N*	PY**	**Patients**	**Hosp**^†^	**%**^‡^	**Freq**^§^	**Hazard**^#^	**Total Rate°**
Month 1	18,695	1,429	1,026	1,049	5.5	1.02	0.72	0.73
Month 2	16,821	1,359	447	451	2.7	1.01	0.33	0.33
Months 3–6	16,319	5,274	1,061	1,208	7.4	0.28	0.20	0.23
Months 7–12	15,361	7,457	871	1,024	5.7	0.20	0.12	0.14
Year 2	14,436	13,642	1,216	1,587	8.4	0.10	0.09	0.12
Year 3	12,917	12,265	1,008	1,316	7.8	0.11	0.08	0.11
Year 4	11,628	11,081	839	1,048	7.2	0.10	0.08	0.10
Year 5	10,514	9,997	747	938	7.1	0.10	0.07	0.09

*N=number surviving to beginning of each time period

**PY=person years accumulated by patients during each period

^†^Hospitalization count in each period

^‡^% gives the proportion of patients hospitalized

^§^Frequency expressed as hospitalizations per patient hospitalized (per month)

^#^Hazard expressed as patients hospitalized per patient year

°Rate expressed as hospitalizations per patient year

**Table 4:** Subsequent hospitalizations for stroke and transient ischemic attack among patients suffering an index ischemic stroke

	**Hospitalizations due to**			
**Time Period**	**Stroke**		**TIA**	
	
	N (%*)	**Rate**^†^	N (%*)	**Rate**^†^
	
Month 1	800 (32.8)	0.56	142 (5.8)	0.10
Month 2	312 (21.4)	0.23	51 (3.5)	0.04
Months 3–6	645 (14.7)	0.12	164 (3.7)	0.03
Months 7–12	433 (8.9)	0.06	152 (3.1)	0.02
Year 2	545 (6.7)	0.04	214 (2.7)	0.02
Year 3	384 (5.6)	0.03	174 (2.5)	0.01
Year 4	344 (5.8)	0.03	102 (1.7)	0.01
Year 5	257 (4.8)	0.03	112 (2.1)	0.01

*Percent of all cause hospitalization

^†^Rate expressed as hospitalizations per patient year
